# Rosai-Dorfman Disease: Case Series and Literature Review

**DOI:** 10.7759/cureus.35193

**Published:** 2023-02-19

**Authors:** Hamzah M Magableh, Hamzh D Jaber, Ahmad M Magableh, Mohammed A Alrabiah, Abdulaziz F Dahhan, Ayman Z Azzam, Tarek Amin

**Affiliations:** 1 College of Medicine, Alfaisal University, Riyadh, SAU; 2 Department of Surgical Oncology, Oncology Center, King Faisal Specialist Hospital and Research Center, Riyadh, SAU; 3 Department of General Surgery, Faculty of Medicine, Alexandria University, Alexandria, EGY

**Keywords:** radiology, surgery, sinus histiocytosis, case report, review, case series, rosai-dorfman

## Abstract

Rosai-Dorfman Disease (RDD), also known as sinus histiocytosis with massive lymphadenopathy, is an uncommon histiocytic condition characterized by massive histopathological aggregation of CD1-a negative, CD68-positive, and S100-positive histiocytes. It was initially described by Destombes in 1965 under the term “adenitis with lipid excess.” However, it is named after Rosai and Dorfman who reported further histopathological features of the disease in 1969. The diagnosis of this non-Langerhans cell histiocytosis can be challenging and requires high clinical suspicion. The diagnostic process usually involves imaging, tissue biopsies, and genetic testing as needed. In this case series, we are presenting three cases of rare disease. Case 2 had both nodal and extranodal forms, which makes this case rarer than cases 1 and 3, which present with extranodal lesions.

## Introduction

Rosai-Dorfman disease (RDD) also known as sinus histiocytosis with massive lymphadenopathy characterized by the overproduction of histocytes immune cells, which accumulates and can invade organs [1]. RDD can be classified into nodal or extranodal. RDD is an R-group histiocytosis according to the 2016 revised histiocytosis classification [1]. Certain diseases, genetic mutations, and viral infections could be associated with RDD [1,2]. The disease associations include familial, immune related, neoplasia associated, and IgG4 related. RDD is usually diagnosed by imaging studies such as computed tomography (CT), magnetic resonance imaging (MRI), and positron emission tomography scans (PET). Frequently, biopsy and immunohistochemical (IHC) studies are required for definitive diagnosis. The characteristic immunohistochemistry of RDD is S100 (+), CD68 (+), and CD1A (-) Treatment options include surgical excision, systematic therapy, and radiation. In this case series, we report three interesting cases of combined subcutaneous and nodal RDD. In this research, different case management will be discussed in light of the pathophysiology of the disease.

## Case presentation

Case 1

**Figure 1:**
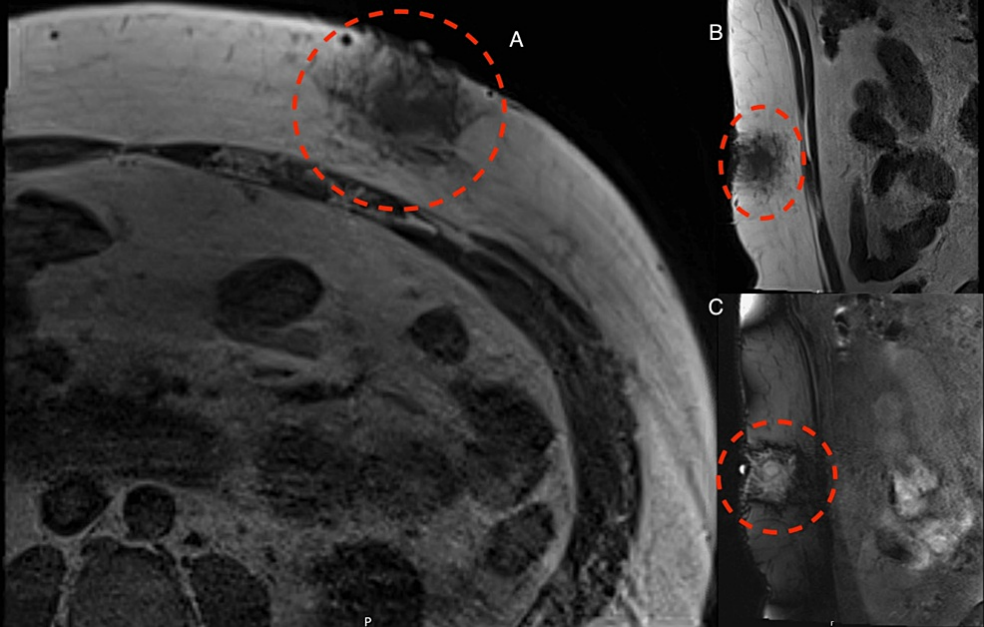
T1- and T2-weighted MRI of abdomen Irregular hyperintense lesions on cross-sectional T1 (A) and sagittal T1 (B). Sagittal T2 (C) with lesion heterogeneity noted The mass is seen involving the left lower anterior abdominal wall with no detectable hemorrhagic components

**Figure 2:**
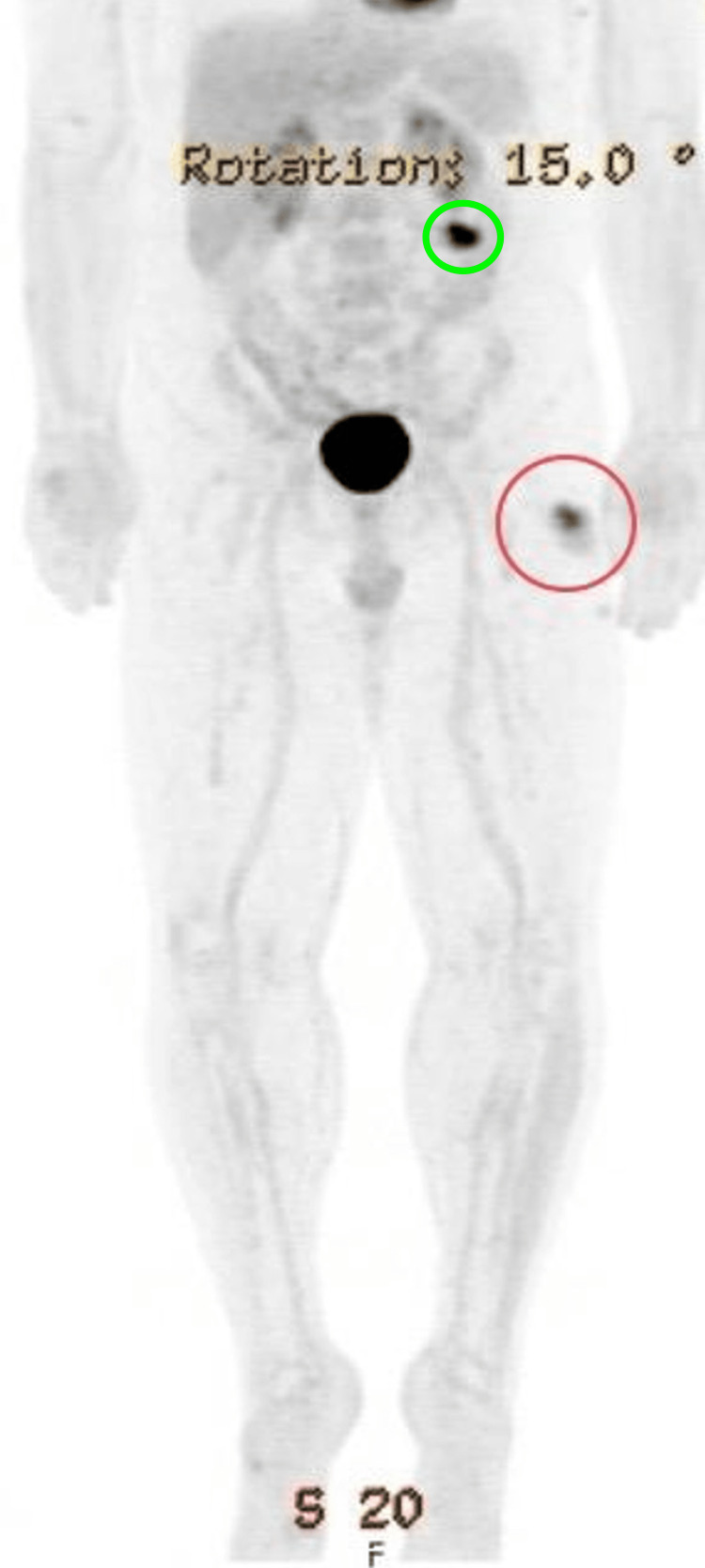
PET scan showing mild fluorodeoxyglucose (FDG) - avid activity in the left upper thigh (red circle) and mild fluorodeoxyglucose (FDG) - avid activity in the left anterior abdominal wall (green circle)

A 46-year-old male presented to our unit complaining of an anterior abdominal wall mass. His MRI showed a lesion with irregular T1 hyperintense, and T2 heterogeneous hyperintense mass involving the left lower anterior abdominal wall with no hemorrhagic components as shown in Figures 1A-1C. An ultrasound-guided biopsy taken from the lesion confirmed RDD. Two other masses were investigated in the left upper back and right lower back respectively. A PET-CT, ordered to investigate the presence of other lesions, showed mild activity in the left lower extremity as shown in Figure 2. Surgical excision of the multiple lesions was the decided mode of treatment.

The following lesions were diagnosed as histiocytic-rich mixed inflammatory infiltrate consistent with RDD. In the left upper thigh, the nodular mass nodular measured 5.5 x 4.0 x 2.0 cm. In the left anterior abdominal wall, the mass was well-defined, tan-white, and firm with central yellow granularity measuring 4.0 x 2.0 x 1.0 cm. Histocytes were immune-positive for CD68, and S100 proteins and negative for CD1A. The examined slides also showed a focal increase of IgG4-positive plasma cells shown in Table 1. The lesions excised from the left upper back and the right lower back were of unremarkable fibrofatty nature and was negative for dysplastic changes.

**Table 1:** Case 1 immunohistochemistry

Marker	S100	Cluster of Differentiation 68 (CD68)	Immunoglobulin G4 (IgG4)	Cluster of Differentiation 1A (CD1A)
	+	+	+	-

The patient had an uneventful post-operative period and was started on chemotherapy and corticosteroids with oral 6-mercaptopurine 50 mg/m^2^ per day and oral Prednisone 40 mg per day for 10 months. The patient was discharged home with outpatient follow-up. The patient had no signs of RDD on follow-up.

Case 2

**Figure 3:**
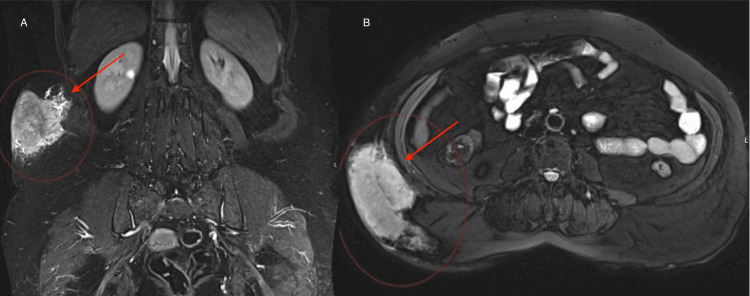
MRI of the abdomen and pelvis showing sagittal (A) and cross-sectional (B) multilobulated subcutaneous soft tissue lesion at the right subcutaneous posterolateral abdominal wall adjacent to the lumbar spine measuring 10 × 5 x 8 cm

A 69-year-old female with a right lumber mass, first detected four years prior to the current visit, complaining of enlargement and escalating pain. On physical examination, the mass was large, mobile, and showed no signs of bone involvement. After referral to our hospital, a pelvic and spine MRI with IV contrast confirmed the presence of a multilobulated subcutaneous soft tissue lesion at the right posterolateral abdominal wall adjacent to the lumbar spine measuring 10 x 5 x 8 cm as shown in Figures 3A, 3B. No metastatic lesions were identified. The patient underwent an ultrasound-guided core biopsy of the right lumbar mass which was confirmatory of RDD. Surgical excision of the mass was performed.

**Figure 4:**
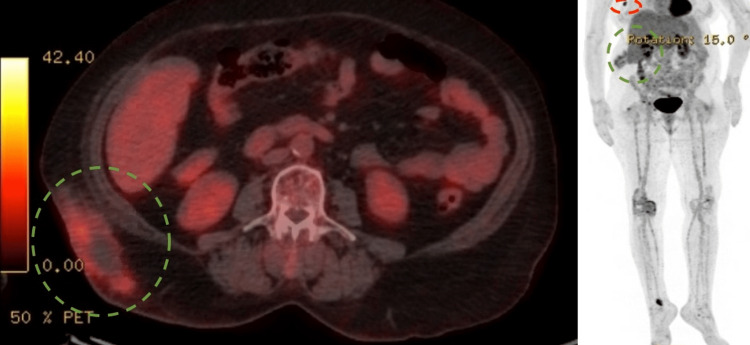
PET-CT scan showing mild heterogeneous soft tissue nodularity with central photopenia and diffuse FDG-avid activity seen in the vicinity of resected right lumbar subcutaneous lesion (green dashed circle) and mild FDG-avid right axillary lesion (red dashed circle)

The postoperative pathology report of the excised mass confirmed a 10 cm right loin lesion consistent with RDD. The lesion is S100 (+), CD1a (-), CD68 (+), CD163 (+), with elevated IgG4 plasma cells shown in Table 2. The tumor involved the skin with its subcutaneous fibroadipose tissue. A PET-CT scan was performed one month post-operatively showing recurrent disease in the right axillary, right inguinal, and distal external iliac nodes Figure 4. All new lesions were biopsied and only the right axillary nodule was identified as RDD., yet the patient is asymptomatic and did not require further treatment.

**Table 2:** Case 2 immunohistochemistry

Marker	S100	Cluster of Differentiation 68 (CD68)	Cluster of Differentiation 163 (CD163)	Cluster of Differentiation 1A (CD1A)	Immunoglobulin G4 (IgG4)
	+	+	+	-	+

Case 3

A 53-year-old female with a history of ovarian cancer diagnosed more than 26 years ago, presented to a local hospital with a four-month history of a left groin 1.5cm lump. PET-CT scan revealed a 1.7 x 0.8 cm subcutaneous mass in the left lower groin. The patient was asymptomatic and was discharged. After one month the patient started having pain so an MRI performed confirmed the presence of a left groin subcutaneous mass measuring 1.2 x 2.3 cm without other lesions or metastasis. Ultrasound-guided biopsy of the mass was performed confirming RDD. Immunohistochemistry results are ALK 1 (-), CD20, and CD3 highlighted in a mixed population of lymphocytes, CK (-), DESMIN (-), S100 (+), SMA (-), CD1a (-) shown in Table 3. Surgical excision of the mass was performed, and the mass had negative margins.

**Table 3:** Case 3 immunohistochemistry

Marker	S100	Cluster of Differentiation 1A (CD1A)	Cytokeratin AE1 /AE3 (CKAE1)	HMB 45	Anaplastic lymphoma kinase (ALK 1)	Cluster of Differentiation 20 (CD20)	Cluster of Differentiation 3 (CD3)	Creatine Kinase (CK)	Desmin	Smooth muscle actin (SMA)	Mart (Antibody)
	+	-	-	-	-	+	+	-	-	-	+

**Figure 5:**
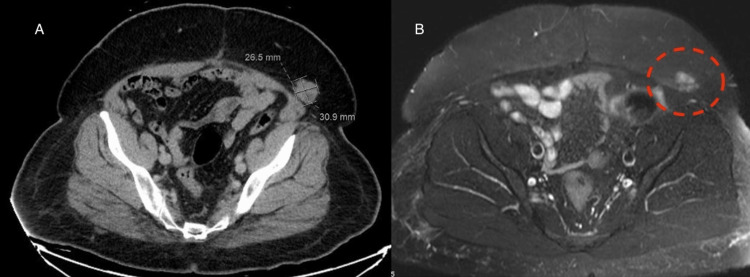
(A) CT scan of the pelvis showing a 2.6 x 3.1 cm mass located in the left groin area and (B) MRI showing left groin subcutaneous soft tissue lesion demonstrating intermediately low signal intensity on T2

**Figure 6:**
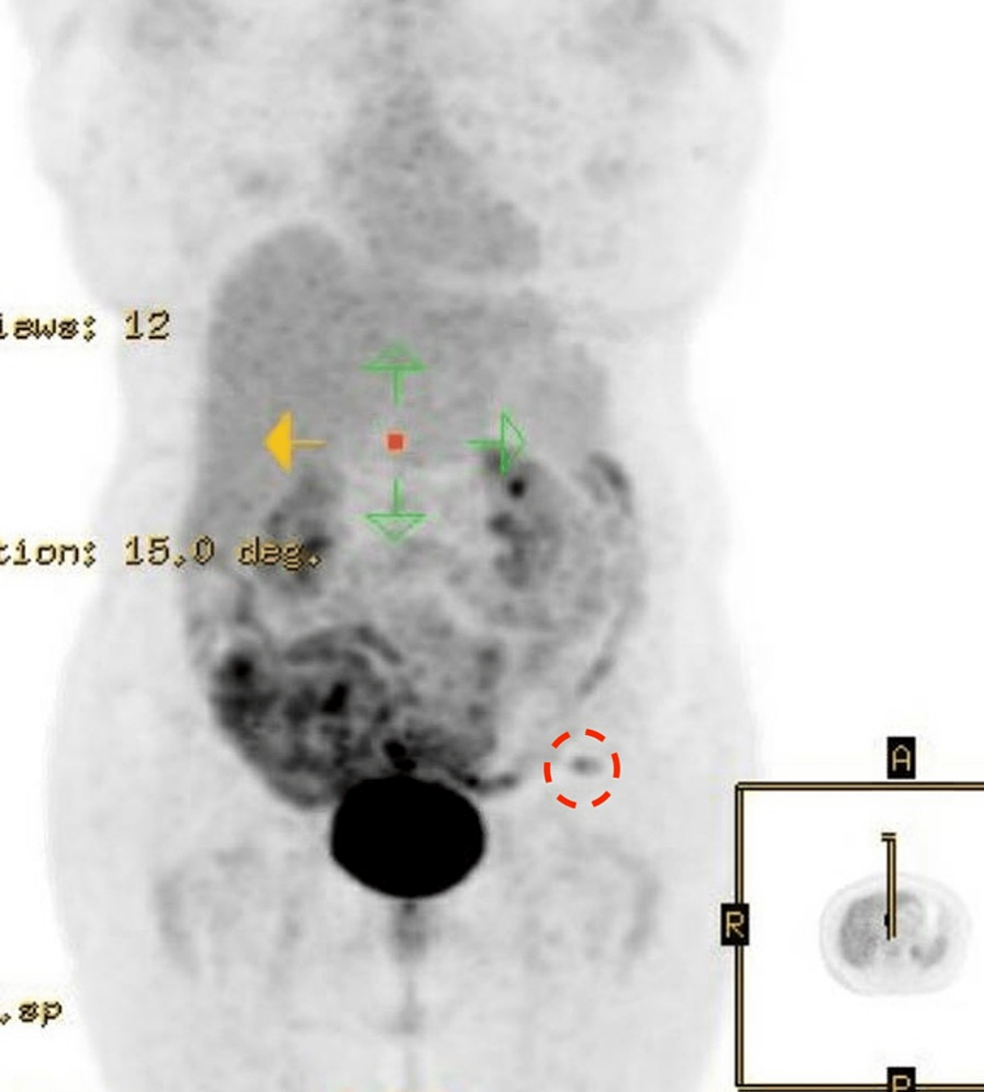
PET scan showing 2 cm left groin mass with mild FDG-avid activity

Two years later, the patient presented to our hospital with a recurrence of the mass in the same region. CT and MRI were performed revealing a left groin mass measuring 2.6 x 3.1 cm as shown in Figures 5A, 5B. A PET-CT showed increased uptake in the mass as shown in Figure 6. Results of an ultrasound-guided biopsy were consistent with RDD S100 (+), AE1/AE3 (-), HMB 45(-), Mart (-), CD1a (-) and CKAE1 (-). The patient underwent surgical excision.

## Discussion

We present a case series of three RDD patients at a tertiary center to report our experience of this rare disease and share the treatment for our patients to contribute to the pool of data and recommendations pertinent to the clinical management of the disease.

RDD is non-Langerhans cell histiocytosis first described in 1969 by Rosai et al. 43% of the cases present as extranodal mass [1]. The prevalence of the disease is 1:200,000 and is more prevalent in children [3]. Some studies have shown that the disease might be related to viral infections such as cytomegalovirus (CMV), human immunodeficiency virus (HIV), and Epstein-Barr virus (EBV) [4]. The literature has also reported the genetic mutation of BRAF-V600E in 23 RDD patients which is a mutation associated with another non-Langerhans cell histiocytosis named Erdheim-Chester disease (ECD) [5]. Other mutations such as SLC29A3, ENT3, KRAS, NRAS, ARAF, and MAP2K1 were also reported [1,2]. The literature reports that 33% of RDD cases had mutually exclusive KRAS and MAP2K1 mutations which can suggest a clonal nature in some RDD subtypes [6]. Next-generation sequencing can be used to determine the genetic mutations that occur in RDD patients; this will offer a tool to further study the pathophysiology of the disease [6].

There are multiple associated diseases in RDD, including familial, neoplasia-associated, immune-related, and IgG4 related. Familial RDD is caused by germline mutations in SLC29A3. SLC29A3 which involves Faisalabad histiocytosis, H syndrome, and pigmented hypertrichotic dermatosis with insulin-dependent diabetes [7]. Neoplasia-associated RDD is RDD that either precedes or appears after lymphoma or myelodysplastic syndrome [8]. Immune-related RDD, which constitutes 10% of RDD cases is associated with systemic lupus erythematous, autoimmune hemolytic anemia, and idiopathic juvenile arthritis [9].

Immunohistochemistry is indispensable when diagnosing RDD, it is usually used to exclude more common entities such as Langerhans cell histiocytosis (LCH), Anaplastic large cell carcinoma (ALCL), and malignant melanoma [10]. A negative CD1a or CD207 staining is essential to exclude LCH; ALCL will reveal positive CD30 staining. Melanoma will demonstrate S100 and HMB45, Milan-A, and SOX10 positivity along with pan-cytokeratin [10]. 

RDD has two distinct clinical forms, extranodal diseases and a nodal form that presents with lymphadenopathy and may include systemic and organ manifestations. The extranodal form being rarer and more prevalent in Asian populations could affect the skin, subcutaneous tissue, the respiratory tract, and the gastrointestinal system [2]. Case 2 had extranodal and nodal involvement, while cases 1 and 3 have the extranodal subcutaneous form only.

Elevated IgG4 plasma cells are seen in some subtypes of RDD, this elevation could be detected either in the lesion, serum titers, or both [11]. RDD was also associated with pancreatic lesions [11]. Evidence suggests that IgG4-RDD may share some pathological features with IgG4-related disease [10]. Case one had IgG4 (+) but did not have any other manifestations. 

CNS involvement in RDD is very rare and reported in less than 5% of all cases with 75% intracranial and 25% spinal [12]. Intracranial RDD may mimic meningioma [12]. Case reports described intracranial involvement with a clinical presentation ranging from headache and episodes of loss of consciousness to diabetes insipidus [2,3]. Bone involvement is associated with the nodal form of the RDD in 5% to 10% of cases, which may predispose to pathological fracture. RDD in a bone usually affects the metaphysis or diaphysis and can manifest as osteolytic or mixed osteolytic and osteoblastic [13].

Our patient in case three had a history of ovarian cancer which we speculate both pathologies may have been related to a KRAS mutation [1]. A case also describes RDD in the ovary mimicking ovarian cancer [14]. We think this should be taken into consideration when managing RDD patients to prevent misdiagnosis.

Asymptomatic RDD is usually observed for spontaneous remission or until the disease becomes symptomatic [15]. Surgical excision was reported as the most effective treatment for the extranodal subcutaneous RDD and was performed for our patients [16]. Systematic therapy mainly includes corticosteroids, Rituximab, 6-Mercaptopurine (6-MP), Cyclophosphamide, and methotrexate [17]. Treatment of choice is selected on a case-by-case basis [1]. Surgical excision was chosen as the first-line treatment for our patients followed by 6-MP in case one. 6-MP was used in case one because it is considered as maintenance therapy after surgical excision, but the optimal dose is not determined [1]. 

## Conclusions

RDD is a rare entity that is challenging in diagnosis and management. RDD can be associated with viral infections, genetic mutations, neoplasia, and immune diseases. RDD has two clinical forms: nodal and extranodal. The extranodal form of the disease is rarer than the nodal form. According to the literature, extrandoal RDD can involve many organ systems. The key diagnosing factor is immunohistochemistry to exclude other pathologies. Treatment should be tailored to each patient to optimize the outcome.
